# Prevalence and predictors of depression among hemodialysis patients: a prospective follow-up study

**DOI:** 10.1186/s12889-019-6796-z

**Published:** 2019-05-09

**Authors:** Amjad Khan, Amer Hayat Khan, Azreen Syazril Adnan, Syed Azhar Syed Sulaiman, Saima Mushtaq

**Affiliations:** 1 Discipline of Clinical Pharmacy, School of Pharmaceutical Sciences, Universiti Sains Malaysia, 11800 Penang, Malaysia; 2 Chronic Kidney Disease Resource Centre, School of Medical Sciences, Health Campus, Universiti Sains Malaysia, 16150 Kubang Kerian, Kelantan Malaysia; 3 Department of Pharmacy, Quaid-i-Azam University, Islamabad, 45320 Pakistan; 4 Health Care Biotechnology Department, Atta ur Rahman School of Applied Biosciences, National University of Science & Technology, Islamabad, 44000 Pakistan; 5 Management Science University, University Drive, Off Persiaran Olahraga, Section 13, 40100 Shah Alam, Selangor Malaysia

**Keywords:** Depression, HADS, Hemodialysis, Hypertension

## Abstract

**Background:**

Even though depression is one of the most common psychiatric disorders, it is under-recognized in hemodialysis (HD) patients. Existing literature does not provide enough information on evaluation of predictors of depression among HD patients. The objective of the current study was to determine the prevalence and predictors of depression among HD patients.

**Methods:**

A multicenter prospective follow-up study. All eligible confirmed hypertensive HD patients who were consecutively enrolled for treatment at the study sites were included in the current study. HADS questionnaire was used to assess the depression level among study participants. Patients with physical and/or cognitive limitations that prevent them from being able to answer questions were excluded.

**Results:**

Two hundred twenty patients were judged eligible and completed questionnaire at the baseline visit. Subsequently, 216 and 213 patients completed questionnaire on second and final follow up respectively. The prevalence of depression among patients at baseline, 2nd visit and final visit was 71.3, 78.2 and 84.9% respectively. The results of regression analysis showed that treatment given to patients at non-governmental organizations (NGO’s) running HD centers (OR = 0.347, *p*-value = 0.039) had statistically significant association with prevalence of depression at final visit.

**Conclusions:**

Depression was prevalent in the current study participants. Negative association observed between depression and hemodialysis therapy at NGO’s running centers signifies patients’ satisfaction and better depression management practices at these centers.

## Background

According to the guidelines of the World Health Organization (WHO), “depression is a common mental disorder, characterized by sadness, loss of interest or pleasure, feelings of guilt or low self-worth, disturbed sleep or appetite, feelings of tiredness and poor concentration” [1]. Among end stage renal disease (ESRD) patient’s depression is one of the most common psychiatric disorders [2]. The prevalence of depression is known to be much higher in HD patients as compared to other individuals of normal population [3]. Like in other chronic disease conditions and in general population, evidence does exist that depression in patients on hemodialysis is associated with mortality [4, 5]. It is under-recognized in HD patients because healthcare providers giving facilities, treatment and routinely work with these patients cannot give attention to control depression due to the nature of their illness [2]. There is a need of regular implementation of screening of depression among this population. Depression and anxiety both are strongly associated with patient’s quality of life (QOL). One study suggests that depression among divorced and widowed women strongly affected patient’s QOL [6].

Different questionnaires have been compiled and tested to investigate and measure the problems of ‘anxiety and depression’ commonly found in ESRD patients, including to those of “Hospital Anxiety and Depression Scale (HADS)” and “Beck’s Depression Inventory (BDI)”, both being properly validated in chronic kidney disease (CKD) patients [7]. The former questionnaire (HADS) was developed with the objective to investigate anxiety and depression associated fresh cases in an adult population. The later (HADS) one is different than the former one as it was developed to address the symptomatic position with respect to anxiety and depression. It is known that HD patients have higher rates of depression prevalence in contrast to the PD patients. The possible reasons are because HD patients usually stay connected with the machine during dialysis procedure which directly affects their daily activities and independence. It has also been reported that among the HD patients suicide rates are much higher. Moreover, it is found that due to the give flexibility and because of limited restrictions in their diet and social activities PD patients live with better quality of life [8–10].

To identify the factors associated with depression and anxiety, there is need of to conduct appropriate longitudinal studies. The instant research work was carried out to determine the contributing action of such factors in causing depression among HD population. Moreover, the expected outcomes of this study will give a comparative information on better management practices of depression at different dialysis facilities.

## Methods

### HADS questionnaire

HADS has been used for numerous studies among HD patients and showed acceptable reliability and validity [7]. Zigmond and Snaith are the original developers of HADS [11]. This scale cannot be used as a clinical diagnostic tool [12]. HADS has many advantages in terms of its interpretability (the results are very easy to interpret), in terms of its acceptability (widely accepted and can be used in a number of populations), in terms of its feasibility (the patients can complete the questionnaire within few minutes, no need of specialized training as the patients themselves can easily completed the questionnaire).

In the current study, we used the official validated Malay version of HADS provided by the original authors of the published Malay version of HADS from the department of psychiatry, Hospital Universiti Sains Malaysia (HUSM) [13].

### Administration of the HADS

The total time required to complete the questionnaire is 2–5 min. Some cautions should be taken into consideration, for instance, the patients should be literate to read it. It can be a reasonable practice for the administrators of the HADS to ask patients first to read it once loudly and then fill it accordingly. HADS is comprised of 14 questions and have two domains: Anxiety (7 items) and depression (7 items). For Anxiety (HADS-A) this gave a specificity and sensitivity of 0.78 and 0.9 respectively. For depression (HADS-D) it gave a specificity and sensitivity of 0.79 and 0.83 respectively [14].

### Study design and setting

This was a prospective follow-up study among HD patients conducted at HUSM and its affiliated dialysis centers. All eligible (> 18 years of age, literate and able to understand Malay) confirmed hypertensive HD patients who were consecutively enrolled for treatment at the study sites from 1st April 2017 to 31st December 2017 were included in the study. Patients with physical and/or cognitive limitations that prevent them from being able to answer questions were excluded.

### Data collection

During the study period, all eligible HD patients who agreed to participate in the study by giving a written consent were asked to self-complete HADS questionnaire at three-time points: i) at baseline visit (initial evaluation), ii) after 3 months’ interval (second follow up) and iii) at 6 months’ interval (third follow up). Enrolled subjects who were unable to participate at the second follow up were not asked to take the questionnaire on third follow up. Using a standardized data collection form, socio-demographic and clinical data were collected from the regularly updated Advanced Dialysis Nephrology Application Network (ADNAN) at study sites. Height, weight and blood pressure were measured during a physical examination. Patient’s interview and data abstraction tool designed by principal investigator based on an input from advisory committee, extensive literature review, hypothetical possible association and nephrologist’s suggestions. At each interview session, the data collector evaluated the questionnaire for completion and asked the subject to provide missing response unless it was intentionally left unchecked.

### Scoring

Grading on HADS questionnaire score sheet was used for scoring of questionnaires. Each question has 4 options; i) yes definitely (3), ii) yes sometimes (2), iii) No, not much (1), iv) No, not at all (0). For items 7 & 10 the scoring is reversed. Scores ranging on HADS from 0 to 7 are considered as non-case, score ranging from 8 to 10 is considered as borderline case and a score of > 11 points were considered as case according to grading system of HADS. For the sake of analysis, we excluded borderline cases and only considered cases and non-cases.

### Statistical analysis

Statistical Package for Social Sciences (SPSS 21) was used for data analysis. Means and standard deviations were calculated for continuous variables, whereas categorical variable are presented as frequencies and percentages. Chi-squared test was used to observe significance between categorical variables. Multivariate logistic regression analysis with the Wald statistical criteria was used to obtain a final model. A *p*-value of < 0.05 was considered statistically significant. Relevant variables with a *p*-value < 0.25 in the univariate analysis were included in the multivariate analysis [15]. We confirmed the correlations among variables entered in the multivariate analysis. The results of multivariate analysis were presented as beta, standard error, *P*-value, adjusted odds ratio and 95% confidence interval. The fit of the model was assessed by Hosmer Lemeshow and overall classification percentage.

## Results

**Fig. 1:**
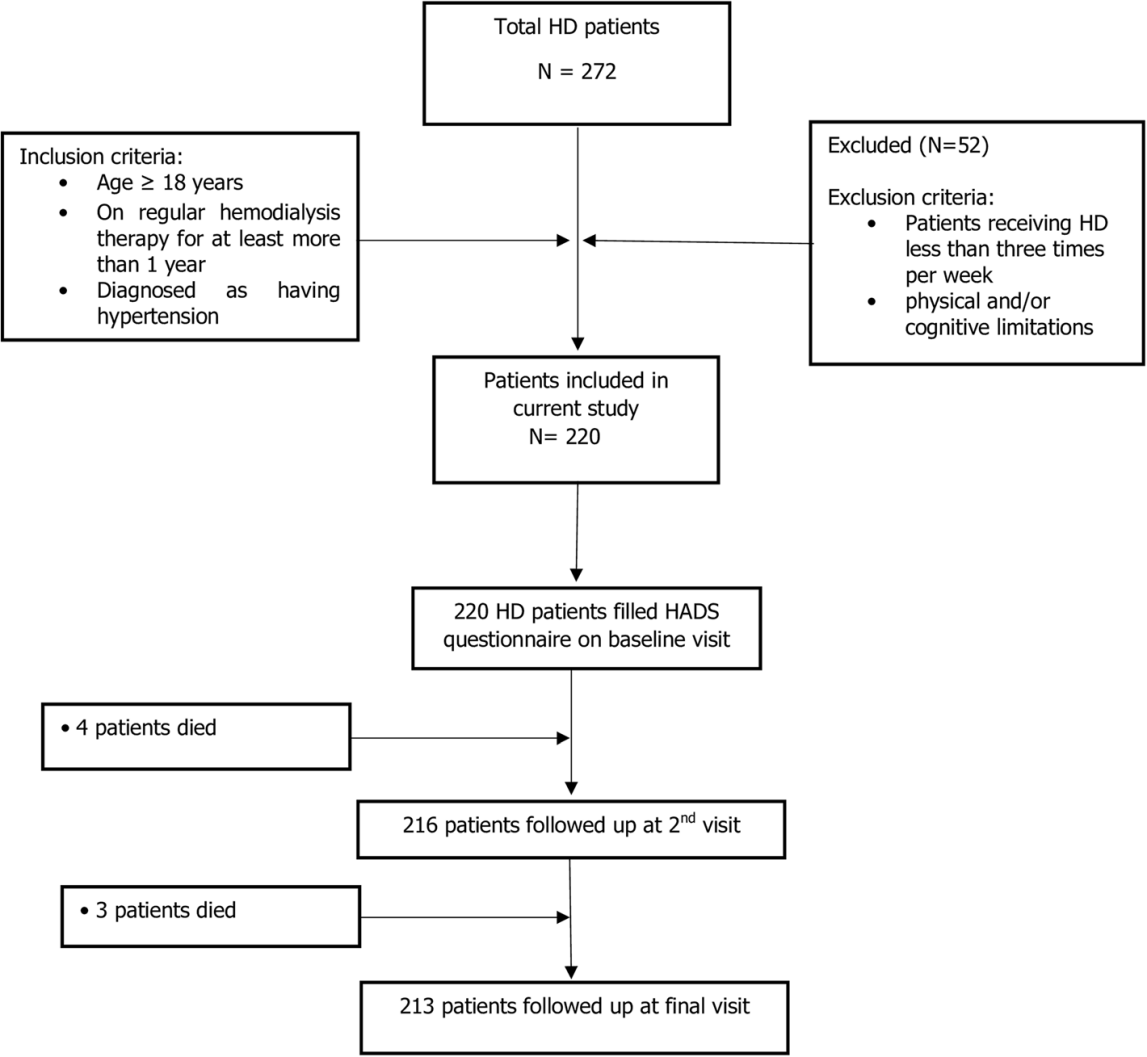
Flow diagram of patient screened, included and evaluated for depression level

During the study recruitment period, a total of 272 HD patients were enrolled for the treatment at the study sites. Fifty-two patients did not meet the eligibility criteria and were excluded. 220 patients were judged eligible and completed questionnaire at the baseline visit. Subsequently, 216 and 213 patients completed questionnaire on second and final follow up respectively (Fig. 1).

### Socio-demographic characteristics of patients evaluated for depression level

The mean patient age was 56.58 ± 11.09 years. The majority of the patients were male (55.5%), 41–60 years old (59.1%), of a normal BMI (67.3%), on dialysis for more than 5 years (36.4%) and suffering from hypertension (91.8%) “Table 1”.

**Table 1:** Sociodemographics and clinical characteristics of patients (*N* = 220)

Variables	No. (%)
Gender	
Female	98 (45.5)
Male	122 (55.5)
Age mean (±SD)	56.58 (± 11.09)
Age group (years)	
< 40	17 (7.7)
41–60	130 (59.1)
> 60	73 (33.2)
BMI mean (±SD)	23.57 (± 4.31)
BMI Classification	
Underweight	12 (5.5)
Normal	148 (67.3)
Overweight	46 (20.9)
Obese	14 (6.4)
Socioeconomic Status	
Low	39 (17.7)
Middle	155 (70.5)
High	26 (11.8)
Education Level	
Uneducated	74 (33.6)
Educated	146 (66.4)
Marital Status	
Single	18 (8.2)
Married	202 (91.8)
Race	
Malay	212 (96.4)
Others	8 (3.6)
Smoking Status	
Current Smoker	73 (33.2)
Non-Smoker	147 (66.8)
Alcohol	
Current drinker	18 (8.2)
Non-drinker	202 (91.8)
Drug Addiction	
Current Drug Addiction	35 (15.9)
No Drug Addiction	185 (84.1)
Employment	
Unemployed	120 (54.5)
Employed	100 (45.5)
Dialysis Years	
1 year	62 (28.2)
2–4 years	78 (35.5)
> 5 years	80 (36.4)
Hemodialysis Centers	
Private	129 (58.6)
NGO	33 (15)
Governmental	58 (26.4)
Vascular access	
Fistula	204 (92.7)
Others	16 (7.3)
Hypertension	
No	18 (8.2)
Yes	202 (91.8)
Diabetes Mellitus	
No	81 (36.8)
Yes	139 (63.2)
Cardiovascular Diseases	
No	185 (84.1)
Yes	35 (15.9)

Other Comorbidities including: Blood clots, depression, asthma, osteoarthritis, pregnancy losses/birth defects and osteoporosis. Low socioeconomic status (≤ RM 2300 or 531 USD), Middle socioeconomic status (RM 2301–5600 or 531–1294 USD) and High socioeconomic status (> RM 5600 or 1294 USD)

*SD* Standard deviation, *BMI* Body Mass Index

### Predictors of prevalence of depression among hemodialysis patients at baseline visit

Table 2 shows that patients gender (OR = 0.690, *p*-value = 0.224), socioeconomic status (high) (OR = 0.500, *p*-value = 0.182), patients receiving treatment at NGO running HD centers (OR = 0.508, *p*-value = 0.105), patients receiving treatment at governmental HD centers (OR = 0.475, *p*-value = 0.030) and multitherapy (OR = 0.659, *p*-value = 0.164) are the variables with *p*-value < 0.25 and will be included in the multivariate analysis.

**Table 2:** Predictors of prevalence of depression among hemodialysis patients at baseline visit (*n* = 220)

Variables	Prevalence of Depression (No. %)		Univariate analysis OR (95% CI)	*P*-value	Multivariate analysis OR (95% CI)	*P*-value
	No	Yes				
Gender						
Female	23 (23.4)	75 (76.6)	Referent		Referent	
Male	40 (32.8)	82 (67.2)	0.690 (0.380–1.254)	0.224	0.742 (0.399–1.383)	0.348
Age (years)						
< 40	4 (23.5)	13 (76.5)	Referent			
41–60	40 (30.8)	90 (69.2)	0.692 (0.213–2.255)	0.542		
> 60	19 (26)	54 (74)	0.874 (0.254–3.012)	0.832		
BMI						
Underweight	4 (33.3)	8 (66.7)	Referent			
Normal	41 (27.7)	107 (72.3)	1.305 (0.373–4.568)	0.677		
Overweight	11 (23.9)	35 (76.1)	1.591 (0.401–6.313)	0.509		
Obese	7 (50)	7 (50)	0.500 (0.102–2.460)	0.394		
Socioeconomic Status						
Low	13 (33.3)	26 (66.7)	Referent		Referent	
Middle	37 (23.9)	118 (76.1)	1.595 (0.745–3.414)	0.230	1.826 (0.816–4.086)	0.143
High	13 (50)	13 (50)	0.500 (0.181–1.382)	0.182	0.570 (0.194–1.677)	0.307
Marital Status						
Single	4 (22.2)	14 (77.8)	Referent			
Married	59 (29.2)	143 (70.8)	0.692 (0.219–2.191)	0.532		
Race						
Malay	62 (29.2)	150 (70.8)	Referent			
Others	1 (12.5)	7 (87.5)	2.893 (0.349–24.011)	0.325		
Smoking status						
Current Smoker	21 (28.8)	52 (71.2)	Referent			
Non-Smoker	42 (28.6)	105 (71.4)	1.010 (0.543–1.877)	0.976		
Alcohol						
Current drinker	6 (33.3)	12 (66.7)	Referent			
Non-drinker	57 (28.2)	145 (71.8)	1.272 (0.456–3.551)	0.646		
Drug Addiction						
Current Drug Addiction	8 (22.9)	27 (77.1)	Referent			
No Drug Addiction	55 (29.7)	130 (70.3)	0.700 (0.299–1.638)	0.411		
Employment						
Unemployed	32 (26.7)	88 (73.3)	Referent			
Employed	31 (31)	69 (69)	0.809 (0.451–1.454)	0.479		
Dialysis Years						
1 year	23 (37.1)	39 (62.9)	Referent			
2–4 years	21 (26.9)	57 (73.1)	1.601 (0.781–3.283)	0.299		
> 5 years	19 (23.8)	61 (76.3)	1.893 (0.914–3.923)	0.686		
Hemodialysis Centers						
Private	29 (22.5)	100 (77.5)	Referent		Referent	
NGO	12 (36.4)	21 (63.6)	0.508 (0.223–1.153)	0.105	0.413 (0.173–0.985)	0.046
Governmental	22 (37.9)	36 (62.1)	0.475 (0.242–0.930)	0.030	0.522 (0.248–1.100)	0.087
Vascular access						
Fistula	59 (28.9)	145 (71.1)	Referent			
Others	4 (25)	12 (75)	1.221 (0.378–3.938)	0.739		
Diabetes Mellitus						
No	23 (28.4)	58 (71.6)	Referent	0.952		
Yes	40 (28.8)	99 (71.2)	0.981 (0.535–1.800)			
Cardiovascular Diseases						
No	55 (29.7)	130 (70.3)	Referent			
Yes	8 (22.9)	27 (77.1)	1.428 (0.611–3.339)	0.411		
Gouty Arthritis						
No	56 (29.3)	135 (70.7)	Referent			
Yes	7 (24.1)	22 (75.9)	1.304 (0.527–3.225)	0.566		
Other Comorbidities						
No	44 (28.2)	112 (71.8)	Referent			
Yes	19 (29.7)	45 (70.3)	0.930 (0.491–1.764)	0.825		
Type Therapy						
Mono-therapy	30 (24.8)	91 (75.2)	Referent		Referent	
Multi-therapy	33 (33.3)	66 (66.7)	0.659 (0.366–1.186)	0.164	0.553 (0.293–1.043)	0.067

Analysis: Univariate and Multivariate binary logistic regression analysis. All variables with *p*-value < 0.25 are included in the multivariate analysis

Low socioeconomic status (≤ RM 2300 or 531 USD), Middle socioeconomic status (RM 2301–5600 or 531–1294 USD) and High socioeconomic status (> RM 5600 or 1294 USD)

*OR* Odds ratio, *CI* confidence interval, *BMI* Body mass index, *NGO* Non-governmental organization

Other comorbidities: Blood clots, depression, asthma, osteoarthritis, pregnancy losses/birth defects and osteoporosis

In Multivariate logistic regression analysis, the only variable which had statistically significant association with prevalence of depression at baseline visit was treatment given to patients at NGO’s running HD centers (OR = 0.413, *p*-value = 0.046) (Table 2).

### Predictors of prevalence of depression among hemodialysis patients at 2nd visit

Table 3 shows that patients’ gender (OR = 0.676, *p*-value = 0.245), treatment at NGO’s running HD centers (OR = 0.519, *p*-value = 0.139), Diabetes (OR = 0.646, *p*-value = 0.219) and multi-therapy (OR = 0.653, *p*-value = 0.198) are the variables with *p*-value < 0.25 and will be included in the multivariate analysis.

**Table 3:** Predictors of prevalence of depression among hemodialysis patients at 2nd visit (*n* = 216)

Variables	Prevalence of Depression (No. %)		Univariate analysis OR (95% CI)	*P*-value	Multivariate analysis OR (95% CI)	*P*-value
	No	Yes				
Gender						
Female	17 (17.3)	81 (82.7)	Referent			
Male	30 (25.4)	88 (74.6)	0.676 (0.351–1.336)	0.245	0.699 (0.357–1.370)	0.297
Age (years)						
< 40	3 (17.6)	14 (82.4)	Referent			
41–60	28 (22)	99 (78)	0.758 (0.203–2.824)	0.679		
> 60	16 (22.2)	56 (77.8)	0.750 (0.192–2.937)	0.680		
BMI						
Underweight	4 (33.3)	8 (66.7)	Referent			
Normal	28 (19.2)	118 (80.8)	2.107 (0.592–7.496)	0.250		
Overweight	10 (22.2)	35 (77.8)	1.750 (0.436–7.032)	0.430		
Obese	5 (38.5)	8 (61.5)	0.800 (0.155–4.123)	0.790		
Socioeconomic Status						
Low	10 (25.6)	29 (74.4)	Referent			
Middle	29 (19.1)	123 (80.9)	1.463 (0.641–3.337)	0.366		
High	8 (32)	17 (68)	0.733 (0.243–2.214)	0.582		
Marital Status						
Single	3 (16.7)	15 (83.3)	Referent			
Married	44 (22.2)	154 (77.8)	0.700 (0.194–2.528)	0.586		
Race						
Malay	47 (22.6)	161 (77.4)	Non-computable			
Others	–	8 (100)		–		
Smoking status						
Current Smoker	15 (21.1)	56 (78.9)	Referent			
Non-Smoker	32 (22.1)	113 (77.9)	0.946 (0.474–1.889)	0.875		
Alcohol						
Current drinker	4 (23.5)	13 (76.5)	Referent			
Non-drinker	43 (21.6)	156 (78.4)	1.116 (0.346–3.598)	0.854		
Drug Addiction						
Current Drug Addiction	7 (20.6)	27 (79.4)	Referent			
No Drug Addiction	40 (22)	142 (78)	0.920 (0.373–2.269)	0.857		
Employment						
Unemployed	23 (19.7)	94 (80.3)	Referent			
Employed	24 (24.2)	75 (75.8)	0.765 (0.400–1.461)	0.417		
Dialysis Years						
1 year	15 (24.6)	46 (75.4)	Referent			
2–4 years	18 (23.7)	58 (76.3)	1.051 (0.478–2.308)	0.902		
> 5 years	14 (17.7)	65 (82.3)	1.514 (0.667–3.439)	0.322		
Hemodialysis Centers						
Private	23 (18.4)	102 (81.6)	Referent		Referent	
NGO	10 (30.3)	23 (69.7)	0.519 (0.217–1.237)	0.139	0.580 (0.238–1.412)	0.580
Governmental	14 (24.1)	44 (75.9)	0.709 (0.334–1.504)	0.370	0.646 (0.295–1.417)	0.276
Vascular access						
Fistula	44 (22)	156 (78)	Referent			
Others	3 (18.8)	13 (81.3)	1.222 (0.333–4.481)	0.762		
Diabetes Mellitus						
No	14 (17.3)	67 (82.7)	Referent		Referent	
Yes	33 (24.4)	102 (75.6)	0.646 (0.322–1.297)	0.219	0.688 (0.335–1.413)	0.309
Cardiovascular Diseases						
No	40 (22)	142 (78)	Referent			
Yes	7 (20.6)	27 (79.4)	1.087 (0.441–2.679)	0.857		
Gouty Arthritis						
No	43 (22.9)	145 (77.1)	Referent			
Yes	4 (14.3)	24 (85.7)	1.779 (0.585–5.409)	0.310		
Other Comorbidities						
No	35 (22.9)	118 (77.1)	Referent			
Yes	12 (19)	51 (81)	1.261 (0.605–2.625)	0.536		
Type Therapy						
Mono-therapy	22 (18.5)	97 (81.5)	Referent		Referent	
Multi-therapy	25 (25.8)	72 (74.2)	0.653 (0.341–1.250)	0.198	0.628 (0.319–1.237)	0.178

Analysis: Univariate and Multivariate binary logistic regression analysis. All variables with *p*-value < 0.25 are included in the multivariate analysis

Low socioeconomic status (≤ RM 2300 or 531 USD), Middle socioeconomic status (RM 2301–5600 or 531–1294 USD) and High socioeconomic status (> RM 5600 or 1294 USD)

*OR* Odds ratio, *CI* confidence interval, *BMI* Body mass index, *NGO* Non-governmental organization

Other comorbidities: Blood clots, depression, asthma, osteoarthritis, pregnancy losses/birth defects and osteoporosis

In multivariate logistic regression analysis, no significant association was found between depression and any sociodemographic and clinical factors (Table 3).

### Predictors of prevalence of depression among hemodialysis patients at final visit

Analysis of prevalence of depression at final visit presented in (Table 4) revealed that BMI (normal) (OR = 4.133, *p*-value = 0.039), BMI (overweight) (OR = 5.333, *p*-value = 0.037), treatment given at NGO’s running HD centers (OR = 0.334, *p*-value = 0.030), treatment given at governmental HD centers (OR = 0.485, *p*-value = 0.105), gouty arthritis (OR = 2.630, *p*-value = 0.203) are the variables with *p*-value < 0.25 and will be included in the multivariate analysis.

**Table 4:** Predictors of prevalence of depression among hemodialysis patients at final visit (*n* = 213)

Variables	Prevalence of Depression (No. %)		Univariate analysis OR (95% CI)	*P*-value	Multivariate analysis OR (95% CI)	*P*-value
	No	Yes				
Gender						
Female	13 (13.7)	82 (86.3)	Referent			
Male	19 (16.1)	99 (83.9)	0.826 (0.385–1.773)	0.624		
Age (years)						
< 40	2 (11.8)	15 (88.2)	Referent			
41–60	20 (15.6)	108 (84.4)	0.720 (0.153–3.394)	0.678		
> 60	10 (14.7)	58 (85.3)	0.773 (0.153–3.911)	0.756		
BMI						
Underweight	4 (40)	6 (60)	Referent		Referent	
Normal	20 (13.9)	124 (86.1)	4.133 (1.071–15.951)	0.039	3.339 (0.833–13.376)	0.089
Overweight	5 (11.1)	40 (88.9)	5.333 (1.110–25.636)	0.037	4.205 (0.834–21.187)	0.082
Obese	< 5	11 (78.6)	2.444 (0.405–14.748)	0.330	1.907 (0.300–12.123)	0.494
Socioeconomic Status						
Low	6 (15.8)	32 (84.2)	Referent			
Middle	20 (13.4)	129 (86.6)	1.209 (0.449–3.258)	0.707		
High	6 (23.1)	20 (76.9)	0.625 (0.177–2.208)	0.465		
Marital Status						
Single	2 (11.1)	16 (88.9)	Referent			
Married	30 (15.4)	165 (84.6)	0.688 (0.150–3.145)	0.629		
Race						
Malay	32 (15.6)	173 (84.4)	Non-computable			
Others	–	8 (100)		–		
Smoking status						
Current Smoker	12 (16.4)	61 (83.6)	Referent			
Non-Smoker	20 (14.3)	120 (85.7)	1.180 (0.541–2.573)	0.677		
Alcohol						
Current drinker	2 (11.1)	16 (88.9)	Referent			
Non-drinker	30 (15.4)	165 (84.6)	0.688 (0.150–3.145)	0.629		
Drug Addiction						
Current Drug Addiction	5 (14.3)	30 (85.7)	Referent			
No Drug Addiction	27 (15.2)	151 (84.8)	0.932 (0.332–2.615)	0.894		
Employment						
Unemployed	17 (14.4)	101 (85.6)	Referent			
Employed	15 (15.8)	80 (84.2)	0.898 (0.422–1.908)	0.779		
Dialysis Years						
1 year	10 (16.9)	49 (83.1)	Referent			
2–4 years	13 (17.6)	61 (82.4)	0.958 (0.387–2.370)	0.925		
> 5 years	9 (11.3)	71 (88.8)	1.610 (0.610–4.253)	0.337		
Hemodialysis Centers						
Private	13 (10.4)	112 (89.6)	Referent		Referent	
NGO	8 (25.8)	23 (74.2)	0.334 (0.124–0.897)	0.030	0.347 (0.127–0.949)	0.039
Governmental	11 (19.3)	46 (80.7)	0.485 (0.203–1.162)	0.105	0.487 (0.196–1.205)	0.120
Vascular access						
Fistula	29 (14.6)	169 (85.4)	Referent			
Others	3 (20)	12 (80)	0.686 (0.182–2.583)	0.578		
Diabetes Mellitus						
No	9 (11.7)	68 (88.3)	Referent			
Yes	23 (16.9)	113 (83.1)	0.650 (0.284–1.487)	0.308		
Cardiovascular Diseases						
No	29 (16.2)	150 (83.8)	Referent			
Yes	3 (8.8)	31 (91.2)	1.998 (0.572–6.973)	0.278		
Gouty Arthritis						
No	30 (16.3)	154 (83.7)	Referent		Referent	
Yes	2 (6.9)	27 (93.1)	2.630 (0.594–11.653)	0.203	2.637 (0.577–12.056)	0.211
Other Comorbidities						
No	24 (16)	126 (84)	Referent			
Yes	8 (19)	55 (87.3)	1.310 (0.554–3.096)	0.539		
Type Therapy						
Mono-therapy	16 (13.8)	100 (86.2)	Referent			
Multi-therapy	16 (16.5)	81 (83.5)	0.810 (0.382–1.719)	0.583		

Analysis: Univariate and Multivariate binary logistic regression analysis. All variables with *p*-value < 0.25 are included in the multivariate analysis

Low socioeconomic status (≤ RM 2300 or 531 USD), Middle socioeconomic status (RM 2301–5600 or 531–1294 USD) and High socioeconomic status (> RM 5600 or 1294 USD)

*OR* Odds ratio, *CI* confidence interval, *BMI* Body mass index, *NGO* Non-governmental organization

Other comorbidities: Blood clots, depression, asthma, osteoarthritis, pregnancy losses/birth defects and osteoporosis

Table 4 shows that in multivariate logistic regression analysis, treatment given to patients at NGO’s running HD centers (OR = 0.347, *p*-value = 0.039) had statistically significant association with prevalence of depression at final visit.

## Discussion

To the best of our knowledge, this is the first follow up study which evaluated the prevalence and factors associated with depression among HD patients in Malaysia. In the current study, 220 eligible patients filled the HADS questionnaire on baseline and 213 filled it at the end of the study.

In our study 157 (71.3%) patients suffered from depression at baseline, 169 (78.2%) on 2nd evaluation and 181 (84.9%) on the final visit respectively. However, the previously published literature has reported a comparatively low rate of depression among HD patients, ranging from 23.3 to 60.5% [2, 16–25].

In our study the rate of depression worsened with the passage of time, a linear increase was found from baseline (71.3%) to final visit (84.9%) among patients. The possible reasons for this finding could be the lifelong dialysis therapy with at least 3 dialysis procedures per week, patients taking too much medicine at once, economic burden on patients and their families and altered family and social relationships. Similar findings were reported in various studies where depression was noted to increase from baseline towards the end of the study period [18, 26, 27]. Keskin et al. revealed that depression is a risk factor for suicidal ideation and the chances of suicide attempts increasing with the severity of depression. Therefore, HD patients should be under regular psychiatric evaluation and all risk factors should be properly evaluated [28]. Depressive symptoms were linearly increasing in a population of chronic HD patients and there was a significant association of poor sleep quality, unemployment, pruritus, hypoalbuminemia and diabetes with depressive symptoms. Women were at increased risk of depression [29].

There is a wealth of evidence that dialysis has negative impact on depression and the severe depression among patients is in turn associated with mortality among these patients. Fifteen large scales studies indicating the significant association of depression with mortality among dialysis patients [30]. Significantly higher mortality risks were observed with depressive symptoms in patients on dialysis therapy in various longitudinal studies that assessed the repeated measurement of depression [31–33]. Studies indicated that depression is associated with initiation of early dialysis treatment [34, 35]. Other studies found relationship of depression with immune and inflammatory responses [36, 37]. Previous studies revealed that poor nutrition and nonadherence to treatment is significantly linked with depression among HD patients [38, 39]. The findings of one other systematic review showed 2-fold risk of dying in patients with depression [40]. Additionally, age is also a risk factor of increased mortality in depressive patients. Findings of another study indicated that in depressive patients with age of 65 years or above, there is 41% higher risk of mortality [41]. Depression is common and serious psychiatric disorder but underrecognized in patients undergoing dialysis therapy. It is reported elsewhere that only one-third of the HD patients with a diagnosis of depression were receiving treatment [42, 43]. Only few observational studies and clinical trials demonstrated the outcomes with pharmacologic and non-pharmacologic therapies in depressive patients [44–48]. Two systematic reviews of antidepressants use in treatment of depression among chronic renal failure patients concluded that the evidence for effectiveness of these drugs is insufficient [49, 50].

In our study, comparable rates of depression were observed among female (86.3%) and male participants (83.9%). In contrast to our finding of no significant association between rate of depression among male and female patients, a study conducted in the University of Michigan, female gender was a significant risk factor for depression [51]. Similar positive association between female gender and high rate of depression among HD patients have been reported elsewhere [52, 53]. On the other hand, in line with our finding, no significant differences were observed in prevalence of depression and life event variables among males and females study participants in a study conducted in Turkey [54]. In our study 86.6% patients with middle socioeconomic status were having depression. In a study conducted elsewhere, an inverse relation was observed between depression and socioeconomic status [55]. Similarly, in another study, poor quality of life and depression was reported in study participants with middle and low socioeconomic status [56]. In another study where authors were interested to determine the association between socioeconomic status and depression among community residents and psychiatric patients, the authors concluded that study subjects holding jobs were more likely to have depression as compared to jobless participants [57].

Of the total 195 married patients, 165 (84.6%) were having depression in the current study. In contradiction to our study findings authors reported that depression was less common in married people which were undergoing dialysis therapy while divorced/widowed patients were at higher risk of depression [52]. Similar results were reported from a study in Taiwan where the status of marriage in HD patients was significantly associated with better quality of life [58]. On the other hand, Kimmel and colleagues reported that rate of depression is higher among divorced and widowed women and depression is associated with patient’s poor quality of life [6]. Supportive and peaceful family environment, happy married life and family support is associated with depression free and better quality of life in chronic HD patients [24]. These findings are in contradiction to the findings of the current study.

Out of the total 140 non-smokers in our study, 85.7% patients were having depression. This is in contradiction to the study findings where authors reported that more than half of the current smokers undergoing dialysis therapy were having depression [59]. Beside in dialysis patients, many epidemiological studies have shown that reciprocal relationship exists between smoking and depression [60–62]. In some studies, it has been reported that health related quality of life (HRQoL) was not improved in patients by smoking cessation [63] and depressed smokers have very less chances to quit smoking [44–66]. Hence, Smoking should be discouraged among HD patients to improve quality of life and to prevent cardiovascular events.

In our study in multivariate logistic regression analysis, treatment given to patients at NGO’s running HD centers (OR = 0.347, *p*-value = 0.039) had statistically significant negative association with prevalence of depression at final visit. Dalrymple et al. found that overall hospitalization rates of HD patients were remarkably higher (15% higher) for those patients which were receiving treatment in for-profit HD facilities as compared with nonprofit dialysis centers [67]. In Malaysia, the government is the main source of funding for new and existing patients on dialysis. There are 3 different sectors i.e. government, NGO’s and private dialysis centers that are providing dialysis treatment to patients in Malaysia. These funds provided by government are not only allocated for government dialysis facilities but also for NGOs running centers, for public pensioners, civil servants and their family members who are undergoing dialysis therapy in private dialysis facilities. Self-funding for dialysis treatment had dropped remarkably from 26% in 2006 to 17% in 2015. Increase in funding from NGO bodies from 12% in 2006 to 15% in 2015 was reported [68]. It is reported that in economically advanced states of Malaysia, patients were taking dialysis treatment in NGOs running centers and in private dialysis centers and the survival rates and quality of life of HD patients were better as compared to public dialysis centers. On the other hand, in states like Sabah, Sarawak, Kelantan and Terengganu 50% of patients were taking dialysis treatment in public sector dialysis facilities [69]. NGOs running programs like Syrian American Medical Society (SAMS) was initiated to help the Syrian patients in refugee camps and northern Syria during the crises in Syria. SAMS was basically a mission of Syrian American nephrologists for the direct observation, to treat psychological disorders and care of dialysis patients which was severely compromised due to destruction of health care facilities, loss of access to dialysis centers, lack of medications and sue to shortage of medical care professionals [70]. But in another study on assessment of ESRD during Syrian crises, the authors found that the aid from inexperienced NGOs and non-renal charities despite of their good will is insufficient and potentially dangerous. Regional and international renal teams should be involved in organizing aid in situations like Syrian crises [71]. A significant improvement in mortality rate over the years and reduce hospitalization rates due to providing adequate dialysis therapy, EPO and iron usage was reported in NGO based dialysis center. Moreover, the free supply of antihypertensive drugs was associated with better control of hypertension and reduced rates of cardiovascular mortality at this NGO funded dialysis facility in south India [72]. Authors of a study reported that Malaysian government reforms to encourage NGOs dialysis facilities and private facilities has brought a transformation and resulted in greatly expanded and an easy access to dialysis patients specially with low socioeconomic status to avail dialysis services [73]. Those dialysis patients who were receiving financial help from NGO’s, hospitals and other funding organizations were less depressed as compared to those who were not [74]. Most notably, the association of depression in NGOs and government sector dialysis centers has never been studied. Further studies are warranted to confirm this finding.

## Strengths and limitations of the study

- This study involved a group of patients from tertiary-level teaching hospital of Malaysia.
- To the best of the authors’ knowledge, this is the first follow up study to assess the prevalence and predictors of depression among hemodialysis patients in a Malaysian setting.
- For determining the factors associated with depression, multivariate analysis was conducted.
- Being a prospective observational study, the findings of the present study need to be interpreted with caution since it is limited to only 6 months follow up.
- Nevertheless, a multicenter study with a large sample size and longer follow up time is needed to confirm the findings of the current study.

## Conclusion

The current study revealed that the negative association of depression with dialysis therapy at NGOs running dialysis facilities is an indication of better depression management practices at these centers. For better management of depression and to enhance the quality of life of HD patients, studies should be carried out on national level in government, private and NGOs running dialysis centers and strategies should be adopted on how to reduce the prevalence of depression where it is more prevalent.

### Study limitations

The findings of the present study need to be interpreted with caution since it is limited to only 6 months follow up. Nevertheless, a multicenter study with a large sample size and longer follow-up time is needed to confirm the findings of the current study. As we have not correlated the depression scores of same individuals assessed on multiple times, our results should be interpreted with the limitation of separate analysis.

## Abbreviations

ADNAN: Advanced Dialysis Nephrology Application Network
BDI: Beck’s Depression Inventory
CKD: Chronic kidney disease
ESRD: End stage renal disease
HADS: Hospital anxiety and depression scale
HD: Hemodialysis
HRQoL: Health related quality of life
HUSM: Hospital University Sains Malaysia
NGOs: Non-governmental organizations
PD: Peritoneal dialysis
QOL: Quality of life
WHO: World health organization
